# A multicentre matched case control study of risk factors for Preeclampsia in healthy women in Pakistan

**DOI:** 10.1186/1472-6874-10-14

**Published:** 2010-04-30

**Authors:** Uzma Shamsi, Juanita Hatcher, Azra Shamsi, Nadeem Zuberi, Zeeshan Qadri, Sarah Saleem

**Affiliations:** 1 Division of Epidemiology/Biostatistics, Department of Community Health Sciences, Aga Khan University, Karachi, Pakistan; 2 Combined Military Hospital, Rawalpindi, Pakistan; 3 Department of Obstetrics/Gynecology, Aga Khan University Hospital AKUH, Karachi, Pakistan

## Abstract

**Background:**

Preeclampsia is one of the leading causes of maternal and perinatal morbidity and mortality world-wide. The risk for developing preeclampsia varies depending on the underlying mechanism. Because the disorder is heterogeneous, the pathogenesis can differ in women with various risk factors. Understanding these mechanisms of disease responsible for preeclampsia as well as risk assessment is still a major challenge. The aim of this study was to determine the risk factors associated with preeclampsia, in healthy women in maternity hospitals of Karachi and Rawalpindi.

**Methods:**

We conducted a hospital based matched case-control study to assess the factors associated with preeclampsia in Karachi and Rawalpindi, from January 2006 to December 2007. 131 hospital-reported cases of PE and 262 controls without history of preeclampsia were enrolled within 3 days of delivery. Cases and controls were matched on the hospital, day of delivery and parity. Potential risk factors for preeclampsia were ascertained during in-person postpartum interviews using a structured questionnaire and by medical record abstraction. Conditional logistic regression was used to estimate matched odds ratios (ORs) and 95% confidence intervals (95% CIs).

**Results:**

In multivariate analysis, women having a family history of hypertension (adjusted OR 2.06, 95% CI; 1.27-3.35), gestational diabetes (adjusted OR 6.57, 95% CI; 1.94 -22.25), pre-gestational diabetes (adjusted OR 7.36, 95% CI; 1.37-33.66) and mental stress during pregnancy (adjusted OR 1.32; 95% CI; 1.19-1.46, for each 5 unit increase in Perceived stress scale score) were at increased risk of preeclampsia. However, high body mass index, maternal age, urinary tract infection, use of condoms prior to index pregnancy and sociodemographic factors were not associated with higher risk of having preeclampsia.

**Conclusions:**

Development of preeclampsia was associated with gestational diabetes, pregestational diabetes, family history of hypertension and mental stress during pregnancy. These factors can be used as a screening tool for preeclampsia prediction. Identification of the above mentioned predictors would enhance the ability to diagnose and monitor women likely to develop preeclampsia before the onset of disease for timely interventions and better maternal and fetal outcomes.

## Background

Preeclampsia (PE) is a pregnancy-specific condition and is associated with high maternal mortality and morbidity as well as risk of perinatal death, preterm birth, and intrauterine growth restriction[1]. It occurs in 4 to 7 per cent of pregnant women worldwide [2]. The etiology of preeclampsia remains unclear despite extensive research. Risk factors for preeclampsia include nulliparity, a family or own history of PE, pre-existing diabetes or increased body mass index, multiple pregnancy, maternal age, renal disease, hypertension or raised blood pressure at booking and chronic autoimmune disease [3]. The rate of preeclampsia has increased worldwide especially in developed countries by 40% between 1990 and 1999 due to an increase in number of older mothers and multiple births, conditions known to increase its risk[4].

Maternal Mortality is extremely high in Pakistan where 1 in 89 women dies of maternal causes with preeclampsia and eclampsia as one of the major causes [5]. As preeclampsia remains a serious and poorly understood complication of pregnancy, we need to identify epidemiological and clinical risk factors to predict it before it threatens the survival of both mother and fetus. The study of risk factors and the underlying evidence base can be used to assess risk of preeclampsia at antenatal booking [6]. There is paucity in studies on preeclampsia and its associated factors in Pakistan; an economically developing country in Asia.

This study was conducted to determine factors associated with PE, in healthy women with single pregnancy in maternity hospitals of Karachi and Rawalpindi. Moreover, this study investigated the association between family history of hypertension and preeclampsia.

## Methods

This matched case control study was conducted between January 2006-December 2007 in six selected maternity hospitals in Karachi and two maternity hospitals in Rawalpindi: Aga Khan University Hospital, Sohbraj Maternity Hospital, Aga Khan Maternity hospital for women, Karimabad, Aga Khan Maternity hospital for Women & Children, Garden, Lady Dufferin Hospital and Jinnah Postgraduate Medical Centre Hospital in Karachi, and Combined Military Hospital and Military Hospital in Rawalpindi.

All pregnant women of any age delivering in any of the above-mentioned hospitals were potential study subjects. We excluded women with past histories of chronic hypertension, and multiple gestations in current pregnancies.

A case was defined as a woman who had given birth during the preceding three days and who, in the ante-natal period or before going into labor, was diagnosed by an obstetrician as being preeclamptic. Preeclampsia was defined as pregnancy-induced hypertension associated with proteinuria. Pregnancy-induced hypertension was defined as new hypertension with blood pressure of 140 mm Hg systolic or diastolic B.P of 90 mm Hg diastolic or greater arising after 20 weeks of gestation in a woman who was normotensive before 20 weeks gestation. Proteinuria was defined as excretion of 300 mg or more of protein in 24 hour urine sample or 1+ or more on dipstick (ICD-9 codes 642E-Fand ICD -10 code O15).

A control was defined as a woman who gave birth during the preceding 3 days and who did not have a diagnosis of preeclampsia. For each case we interviewed two controls who delivered on the same day in the same hospital matched on parity. In total 131 cases and 232 controls were interviewed. The hospital's register was surveyed each day by the study team members to identify all preeclamptic cases, and potential controls. Prior to the interview, informed consent was obtained from all study participants. Women with preexisting chronic hypertension, defined as BP greater than 140/90 mmHg before pregnancy or before 20 weeks' gestation, were excluded.

The data collection was conducted on the post delivery ward by the specialized doctors using a structured questionnaire. Women with singleton gestations delivered after 20 weeks were eligible for inclusion. They were interviewed at enrollment and within 3 days of delivery. The questionnaire included information regarding sociodemographic characteristics, antenatal history and family history of hypertension and diabetes apart from other covariates.

Potential risk factors were selected on the basis of literature review and biological plausibility for an association with both the exposure and outcome. The covariates included in the analysis were maternal age, maternal smoking, gestational diabetes, diabetes mellitus, stress measured by Perceived Stress Score PSS, sociodemographic status, age at menarche, body mass index, urine tract infections, past history of PE, family history of hypertension, diabetes mellitus, use of condoms, age of husband, sex of the baby, blood group and RH factor. Diabetes mellitus and gestational diabetes were self reported by the participants and verified by the medical records. Perceived Stress Scale (PSS) used was a 10-item measure of stressful situations during the past month [7]. Items are scored on a five-point scale from 0 to 4; the total score provides a global measurement of the extent to which an individual feels overwhelmed. Total scores range from 0 to 40; higher scores indicate greater perceived stress. As we don't have the data on incidence or prevalence of PE in Pakistan therefore the prevalence of various risk factors for pre-eclampsia was ascertained through literature search. In the sample size calculation, we assumed the prevalence of the various risk factors amongst the control group to be in the range of 11-72%. The prevalence of Diabetes in Pakistan is 11%. With low prevalence of disease as well as time and financial constraints, we took the option of taking a 1:2 ratio between cases and controls to increase the power of the study. Thence in order to be able to detect an odds ratio of at least 2 with a power of 80%, at a significance level of 5%, we calculated a sample size of 187 cases and 374 controls. {NCSS statistical software & power analysis sample size PASS}.

All data analyses were performed using SAS, version 9.1, for windows (SAS Institute Inc. North Carolina) and SPSS 16. To identify the factors associated with PE, univariate matched analysis was done by comparing the cases and controls (1 case: 2 controls) for each variable of interest and crude matched odds ratio and their 95% confidence intervals along with p values were calculated. In multivariable analysis, matched analysis in logistic regression was performed to identify associated factors of PE while adjusting for other variables. Finally any variable with p-value > 0.05, not a confounder or interacting with other variables were removed from the model to obtain a parsimonious and biologically meaningful model that best explains the phenomena of PE.

The study was approved by the Ethical Review Board of Aga Khan University Hospital.

## Results

The sociodemographic, obstetric and antenatal characteristics of all participants are presented in table 1. The actual sample size achieved was a total of 131 cases matched on parity, day of delivery and hospital with 262 controls participated in the study of preeclampsia risk factors. There were 194 nulliparous women and 199 parous women with single pregnancy. No significant differences were observed between cases and controls with regard to maternal age, age at menarche, maternal education, socioeconomic status measured by ownership of house, number of rooms in household, number of household members, husband's occupation.

**Table 1:** Table showing demographic, socio-economic and obstetrical characteristics of cases and controls with preeclampsia (PE) among women in maternity hospitals of Karachi and Rawalpindi (January 2006-December 2007)

Variables	Cases (131) n (%)	Controls (262) n (%)	P-value
**Parity**			
Nulliparous women	64 (48.9)	130 (49.6)	0.915
Parous women	67 (51.1)	132 (50.4)	
**Mean maternal age (years)**			
≤ 18	5 (3.8)	12 (4.6)	0.935
19-34	114 (87.0)	226 (86.6)	
≥ 35	12 (9.2)	23 (8.8)	
**Age at menarche**	13.0 ± 1.2	13.0 ± 1.3	0.476
**Maternal Smoking**	5 (3.8)	16 (6.1)	0.42
**Maternal education**			
Less than 8 years	41 (31.3)	69 (27.2)	0.696
8-12years	64 (48.9)	131 (51.6)	
More than 12 years	26 (19.8)	54 (21.3)	
**Ownership Status**			
Owned	91 (69.5)	160 (61.1)	0.102
Rented	40 (30.5)	102 (38.9)	
**Number of rooms in household**			
≤ 3	73 (55.7)	145 (55.3)	0.943
> 3	58 (44.3)	117 (44.7)	
**Mean number of household members**			
≤ 6	63 (48.1)	147 (56.1)	0.133
>6	68 (51.9)	115 (43.9)	
**Husband's occupation**			
Private job	42 (32.1)	98 (37.4)	0.296
Government job	50 (38.2)	108 (41.2)	
Business	29 (22.1)	44 (16.8)	
Others	10 (7.6)	12 (4.6)	
**Monthly household income**			
>10000	45 (34.4)	118 (45.2)	0.034
4001-10000	65 (49.6)	120 (46.0)	
≤ 4000	21 (16.0)	23 (8.8)	
**Gestational diabetes**	16 (12.4)	05 (1.9)	< 0.001
**Urinary tract infection**	39 (31.0)	47 (18.5)	0.006
**Pregestational Diabetes Mellitus**	07 (5.6)	02 (0.8)	0.004
**Family History of DM**	56 (43.8)	75 (29.6)	0.006
**Family History of Hypertension**	76 (58.9)	100 (38.5)	< 0.001
Perceived Stress Score*	22 ± 6.1	20 ± 6.2	0.002
Husband's Age*	33.5 ± 12.9	33.5 ± 13.5	0.981
**Condom use around the time of conception**	17 (13.5)	34 (13.7)	0.954
Height* (centimeters)	150.8 ± 22.3	149.5 ± 23.5	0.592
Weight*(kilograms)	57.3 ± 15.8	55.0 ± 12.2	0.002
**BMI**‡			
<18.5	18 (13.7)	37 (14.1)	0.502
18.5-24.9	78 (59.5)	171 (65.3)	
25-29.9	23 (17.6)	39 (14.9)	
≥ 30	12 (9.2)	15 (5.7)	
Gestational age* (weeks)	36.2 ± 2.2	37.0 ± 1.2	<0.001
**Blood Group**			
A+/A-	30 (24)	47 (18.4)	0.326
B+/B-	42 (33.6)	75 (29.4)	
AB+/AB-	11 (8.8)	30 (11.8)	
O+/O-	42 (33.6)	103 (40.4)	
**Rh Factor positive**	120 (96)	231 (90.6)	0.067
**Rh factor negative**	5 (4)	24 (9.4)	
**Sex of Baby**			
Male	77 (61.1)	151 (58.5)	0.628
Female	49 (38.9)	107 (41.5)	

*Plus-minus values are means ± SD.

‡BMI = body mass index, calculated by dividing weight in kilograms with height in meters squared.

Gestational diabetes was higher among cases (12.4%) as compared to controls (1.9%). Urine tract infections during pregnancy were higher among cases compared to controls (31% among cases and 18.5% among controls). Pregestational diabetes was higher among cases (5.6%) compared to controls (0.8%). Family history of diabetes among first relatives was 43.8% among cases and 29.6% among controls. Family history of hypertension among cases was 58.9% as compared to 38.5% among controls. Mean Perceived stress score was 22 among cases as compared to 20 among controls. The mean prepregnancy weight for cases was 57.3 kg and for controls it was 55 kgs. There were no differences among cases and controls with regard to age of husbands, condom use around conception, sex of baby, maternal height, BMI, Blood group and Rh factor.

Referring to table 2, the significant variables in the final model were history of gestational diabetes, pregestational diabetes mellitus, family history of hypertension and perceived stress score. After adjusting for the effect of other variables in the model it was found that gestational diabetes was independently associated with preeclampsia. Women with gestational diabetes were at greater odds of having preeclampsia as compared to those who had no gestational diabetes (OR = 6.57, CI; 1.94-22.25).

**Table 2:** Table based on multivariable analysis showing the association of independent variables with PE risk among women in maternity hospitals of Karachi and Rawalpindi (January 2006-December 2007)

Variables	Matched unadjusted OR(95% CI)	Matched adjusted OR(95%CI)
**Gestational diabetes**	6.40 (2.34, 17.4)	6.57 (1.94, 22.25)
**Urinary tract infection**	2.0 (1.21, 3.49)	NS
**Pregestational Diabetes Mellitus**	6.56 (1.36, 31.7)	7.36 (1.37, 33.66)
**Family History of DM**	1.88 (1.18, 3.00)	NS
**Family History of Hypertension**	2.24 (1.45, 3.46)	2.06 (1.27, 3.35)
Perceived Stress Score* (for every 5 unit change)	1.33 (1.21, 1.46)	1.32 (1.19, 1.46)
Husband's Age*	1.0 (0.98, 1.02)	NS
**Condom use around the time of conception**	1.02 (0.50, 2.11)	NS
Height* (centimeters)	1.01 (.98, 1.05)	NS
Weight*(kilograms)	1.02 (1.01, 1.04)	NS
**BMI**	1.05 (1.00, 1.11)	NS
**Sex of Baby** Male Female	1.15 (0.74, 1.78)1	NS
**Monthly Household Income**		
> 10000	0.35 (0.16 " 0.76)	NS
4001 " 10000	0.54 (0.26 " 1.13)	NS
< 4000	1	
		

* Plus-minus values are means ± SD.

NS Nonsignificant

Multivariate adjusted model was adjusted for maternal age, socioeconomic status measured by ownership of house, number of household members and monthly household income, urine tract infection during pregnancy, family history of diabetes mellitus,

maternal weight, Rh factor.

Similarly history of pre-gestational diabetes was independently associated with preeclampsia. Women with a history of pre-gestational diabetes were at greater odds of having preeclampsia as compared to women without pre-gestational diabetes (OR = 7.36, CI; 1.37-33.66).

Our study showed that the family history of hypertension was an important risk factor for preeclampsia. Cases were 2.3 times more likely to have a positive family history of hypertension as compared to controls, while keeping no family history of hypertension as the reference category (OR = 2.06, CI; 1.27-3.35).

Mental stress during pregnancy, measured by Perceived Stress Scale, was also associated with increased risk of PE (OR = 1.32; CI; 1.19-1.46). For each 5 unit increase in the stress score the risk of preeclampsia increased by 1.32. There was no collinearity between variables included in the final model.

## Discussion

This is the first report of a hospital based case control study to determine preeclampsia risk factors among women in Karachi and Rawalpindi. Participants provided information on all the potential risk factors during postpartum interviews.

Our results showed that gestational diabetes mellitus (GDM) was associated with the subsequent development of preeclampsia. The result of our study showing a relationship between preeclampsia and diabetes is also consistent with previous findings [6,8-13]. Gestational diabetes is independently and significantly associated with an increased risk of preeclampsia and an even minor degree of glucose intolerance is associated with preeclampsia [14-16]. Similarly there was a positive association between pregestational diabetes and preeclampsia risk. This finding is also consistently seen in previous studies [15,17-20]. This association is based on a small number of cases and control subjects and the confidence interval is wide.

The findings from our study are biologically plausible for reason that epidemiological and clinical data document a close association between insulin resistance, type 2 diabetes, and hypertension. Hyperinsulinemia has been shown to stimulate the proliferation of vascular smooth muscle cells, enhance acute sympathetic nervous system activity and modify transmembrane cation transport, as well as renal sodium retention and associated endothelial dysfunction. All of these alterations may contribute to blood pressure elevations [21,22].

Our observation of a positive association between family history of chronic hypertension and risk of preeclampsia is consistent with several previous reports [23-25]. These studies reported an increase in risk of preeclampsia with positive family history of chronic hypertension. Our results suggest that family history of hypertension reflects genetic and behavioral factors whereby women may be predisposed to an increased preeclampsia risk. Family history of chronic hypertension is a proxy measure for hereditary factors as well as common environmental or behavioral exposures that may underlie preeclampsia risk [26,27]. To the best of our knowledge, this is the first analysis in Pakistan to assess the effects of family history of chronic hypertension on preeclampsia risk.

Women's family history of chronic hypertension is an important and easy to acquire clinical risk marker of preeclampsia compared to the biochemical markers. During the past 100 years numerous clinical, biophysical, and biochemical tests have been suggested to identify women who are at increased risk for future development of preeclampsia. Unfortunately, these biomarkers have limited sensitivity and are expensive enough for our women to be clinically useful for the prediction of preeclampsia in our setting. The family history of hypertension questions can be used as an inexpensive and feasible screening tool to identify pregnant women in a developing country like Pakistan to monitor the signs of preeclampsia during early pregnancy.

Our study showed an association of mental stress during pregnancy and development of preeclampsia. Depression and anxiety in early pregnancy are associated with risk for preeclampsia, a risk further increased if associated with vaginosis [28] Stressful work environment and stressful home environment are also associated with preeclampsia [29]. The prenatal stress alters maternal physiology and immune function in a manner consistent with increased risk of pregnancy complications such as preeclampsia and premature labor [30]. In another study work-related psychosocial strain increased the risk of PE [31]. Vasoconstriction in preeclampsia may develop early in pregnancy. Indeed, increased uterine artery resistance in maternal anxiety could be a primary manifestation or even the cause of preeclampsia [32,33].

Several limitations must be considered when interpreting the results from our study. First of all recall bias and inability to establish temporality between preeclampsia and certain variables are inherent due to the case control study design. We cannot exclude the possibility that our results could be partially confounded by unidentified risk factors. Our study would have been strengthened by a larger number of preeclamptic women, particularly those with histories of exposures like extremes of age, condom use, women with change in paternity. Prepregnancy weights and body mass index (BMI) were assessed by subtracting the average weight gain from the full term maternal weight which might not be a good proxy measure. There is certainly underreporting of smoking, condom use and therefore we could not establish a relationship between them and preeclampsia risk. Dietary and other lifestyle changes relationship with preeclampsia not analyzed in this study. This being a hospital based study; the results may not be applicable to the general population at large. We could not establish a relationship between certain factors like condom use and change of paternity because of their low prevalence of these exposures in our population. The risk factors of preeclampsia may be different in early vs. late onset of preeclampsia but we did not record the gestational age of PE diagnosis and therefore we were unable to categorize PE in early vs. late onset.

The study was conducted in accordance with the American College of Obstetricians & Gynecologists (ACOG) criteria and ICD 9& 10 for preeclampsia. The American College of Obstetricians & Gynecologists (ACOG) definition for preeclampsia was used to avoid misclassification of preeclampsia with gestational hypertension as well as superimposed preeclampsia [34]. The participation rate for both control and cases subjects was 100%, so there was no selection bias. The data collected was mainly by classified doctors in gynecological and Obstetrics Departments with rich clinical as well as research experience and therefore, the quality of data collected was good and accurate.

Some of the predictors like Diabetes and GDM are modifiable and preventable and others like family history of preeclampsia even if not amenable to change but still are useful in identification of women at high risk who require extra vigilance. If greater awareness of the associated risk factors leads to earlier diagnosis and improved management, there may be scope for reducing a proportion of the morbidity and mortality from preeclampsia.

## Conclusion

Factors like gestational diabetes, pregestational diabetes, family history of hypertension and mental stress during pregnancy can be used as screening tools for preeclampsia prediction. These factors can help identify pregnant women who need closer monitoring for the signs of preeclampsia during early pregnancy. Every woman during the antenatal visit should also be asked for the family history hypertension in order to better estimate the possible risk of developing preeclampsia. These risk factors should be considered for the designs of future studies of preeclampsia in our population of Pakistani women.

## Competing interests

The authors declare that they have no competing interests.

## Authors' contributions

US was the PI of the study who developed the protocol as a part of her Master's thesis. She developed the design of the study, carried out the statistical analyses, participated in interpreting results, and drafted the manuscript. JH was the supervisor and she supervised the protocol, participated in interpreting results. NZ was the subject expert and gave his expertise during the study design phase. AS participated in the design of the study, coordinated the data collection in Rawalpindi hospitals and contributed to the manuscript writing. ZQ supervised the data entry and data editing along with statistical expertise. SS provided support for the protocol writing, participated in interpreting results and manuscript writing. All authors have reviewed the final manuscript.

## Pre-publication history

The pre-publication history for this paper can be accessed here:

http://www.biomedcentral.com/1472-6874/10/14/prepub
